# Combining structure and genomics to understand antimicrobial resistance

**DOI:** 10.1016/j.csbj.2020.10.017

**Published:** 2020-10-29

**Authors:** Tanushree Tunstall, Stephanie Portelli, Jody Phelan, Taane G. Clark, David B. Ascher, Nicholas Furnham

**Affiliations:** a Department of Infection Biology, London School of Hygiene and Tropical Medicine, Keppel Street, London WC1E 7HT, UK; b Computational Biology and Clinical Informatics, Baker Heart and Diabetes Institute, Australia; c Structural Biology and Bioinformatics, Department of Biochemistry and Molecular Biology, Bio21 Institute, University of Melbourne, Australia; d Department of Infectious Disease Epidemiology, London School of Hygiene and Tropical Medicine, Keppel Street, London WC1E 7HT, UK

**Keywords:** Structural bioinformatics, Machine learning, Antimicrobial resistance, Tuberculosis, Genome wide association studies, Pathogen surveillance

## Abstract

Antimicrobials against bacterial, viral and parasitic pathogens have transformed human and animal health. Nevertheless, their widespread use (and misuse) has led to the emergence of antimicrobial resistance (AMR) which poses a potentially catastrophic threat to public health and animal husbandry. There are several routes, both intrinsic and acquired, by which AMR can develop. One major route is through non-synonymous single nucleotide polymorphisms (nsSNPs) in coding regions. Large scale genomic studies using high-throughput sequencing data have provided powerful new ways to rapidly detect and respond to such genetic mutations linked to AMR. However, these studies are limited in their mechanistic insight. Computational tools can rapidly and inexpensively evaluate the effect of mutations on protein function and evolution. Subsequent insights can then inform experimental studies, and direct existing or new computational methods. Here we review a range of sequence and structure-based computational tools, focussing on tools successfully used to investigate mutational effect on drug targets in clinically important pathogens, particularly *Mycobacterium tuberculosis*. Combining genomic results with the biophysical effects of mutations can help reveal the molecular basis and consequences of resistance development. Furthermore, we summarise how the application of such a mechanistic understanding of drug resistance can be applied to limit the impact of AMR.

## 1 Introduction

### 1.1 Antimicrobial resistance (AMR)

Drugs against bacterial, viral and parasitic pathogens have truly revolutionised modern medicine, transforming human health and saving millions of lives. This transformation, however, is under threat due to emerging and widespread resistance to these drugs [1]. This threat is termed antimicrobial resistance (AMR), and is a natural and expected consequence of the Darwinian principle of “survival of the fittest”. Almost all antimicrobial drugs have seen resistance arise within 5–10 years of their introduction [2]. The consequences of AMR pose a catastrophic public health threat, responsible for over 700,000 annual deaths [3], prolonged hospital stays, poor disease outcome, less effective treatments, and potentially untreatable diseases. Considering antibiotic resistance alone, the toll is predicted to rise above 10 million deaths per year by 2050 if left unchecked. The associated global economic burden is estimated at 100 trillion USD [3].

The disease burden of AMR has been accelerated by the overuse and misuse of antimicrobials in health, animal and agricultural industries. This burden is further compounded by a lack of market incentives for antimicrobial drug development [3]. Nearly all major infectious diseases are affected by either prevailing or emerging resistance. For example, it is estimated that people with MRSA (Methicillin-Resistant *Staphylococcus aureus*) are 64% more likely to die than people with a non-resistant form of the infection [1]. Similarly, resistance to artemisinin-based combination therapy, the first-line treatment for malaria caused by *Plasmodium falciparum (P. falciparum)*, has been confirmed in 5 countries in the Greater Mekong Region in 2016 [1]. Likewise, in 2010, an estimated 7–15% patients starting antiretroviral therapy (ART) in developing countries had drug-resistant HIV, with up to 40% resistance observed in patients re-starting treatment [1].

Tuberculosis (TB), caused by *Mycobacterium tuberculosis* (*Mtb*), is a major global health problem, with increasing drug resistance making disease control difficult [4]. In 2017, 558,000 cases of rifampicin resistant TB were reported, among which 82% had additional resistance to isoniazid, leading to multidrug-resistant TB (MDR-TB). Among these MDR cases, ~9% cases were further resistant to one fluoroquinolone and one injectable 2nd line drug, leading to extensively drug resistant TB (XDR-TB) [5], [6].

Resistance is attributed to multiple factors including selective pressure on *Mtb* from repeated exposure to the same antibiotic, a lack of access to new therapies, and patient non-compliance due to long treatment regimens and drug toxicity effects [7], [8]. Both phenotypic and genotypic routes are involved in the development of *Mtb* resistance. While epigenetic changes and post transcriptional modifications drive the phenotypic route to resistance [9], [10], the genetic route is chiefly acquired via accumulation of mutations in the absence of horizontal gene transfer. Resistance-associated point mutations have been described across all anti-TB drugs, including newer ones (fluoroquinolones, bedaquiline) [11], [12].

### 1.2 Drivers of AMR

The drivers of AMR can be both intrinsic or acquired. Intrinsic resistance refers to the innate mechanisms present within microbes to combat the action of drugs, and is considered to be independent of previous drug exposure. Intrinsic mechanisms include:

(i) the presence of an additional impermeable outer membrane in Gram negative bacteria making them naturally resistant to antibiotics that target cell wall synthesis such as vancomycin [13].
(ii) the presence of enzymes that either prevent drug binding within an organism, or destroy the drug. An example of the former is the low affinity binding by Gram positive bacteria of penicillin-binding proteins (PBPs) required for the synthesis of peptidoglycan in the cell wall, thus making them naturally resistant to the β-lactam antibiotic aztreonam. An example of the latter is the production of β-lactamase by Gram negative bacteria which destroy β-lactam antibiotics before they can reach their PBP targets [14].
(iii) the presence of multi-drug efflux pumps, which are complex bacterial molecular machines capable of removing drugs and toxic compounds out of the cell. For example, efflux mediated drug resistance in tetracycline is mediated by the Tet efflux pumps which use proton exchange as its energy source to expel the antibiotic [15].
(iv) the lack of enzymes or metabolic pathways in aerobic bacteria to chemically reduce the drug metronidazole to its active form [13].
(v) the co-evolution of microbes with their surroundings containing a variety of toxic and benign molecules and compounds, which is commonly observed in environmental microbes. For example, the soil bacteria actinomycetes harbours an intrinsic ‘resistome’ to the many antibiotics it produces [16], [17].
(vi) the phenomenon of bacterial persistence, notably observed in asymptomatic and chronic infections such as typhoid and TB. Persisters are a sub population of antibiotic tolerant cells that exhibit low metabolic activity and arrested growth, contributing to increased drug tolerance and resistance [18].

Acquired drug resistance is typically driven by genetic variation including point mutations (missense mutations or non-synonymous single nucleotide polymorphisms; nsSNPs) and insertions/deletions (INDELs) such as frameshift mutations. Such mutations can alter drug activation, binding affinity and permeability, efflux pump activity, and biofilm formation [19]. Furthermore, a common and prominent mechanism called horizontal gene transfer (HGT) or lateral gene transfer (LGT) has been a significant cause of widespread drug resistance. HGT/LGT is found almost exclusively in bacteria where resistance conferring genes are transferred between bacterial species [20], [21].

Despite the two distinct routes of resistance, intrinsic mechanisms may be driven by adaptive/acquired routes. For example the efficacy of drug efflux pumps in *Mtb* are modulated by SNP mutations [22], [23]. The drivers of AMR and the various mechanisms beyond point mutations (which forms the focus of this review) have been extensively reviewed elsewhere: antibiotic resistance [13], [14], antifungal resistance [24], [25], [26], antiviral resistance [27], [28] and antiparasitic drug resistance [29], [30], [31].

### 1.3 Point mutations linked to AMR

A major route to AMR is driven by point mutations. For example, in *Mtb,* mutations in several genes have been associated with resistance to rifampicin (*rpoB),* isoniazid (*katG,* *inhA* and *ahpC),* streptomycin *(gidB, rrs* and *rpsL),* pyrazinamide (*pncA)*, ethambutol (*embB)* and fluroquinolone (*gyrA* and *gyrB)*. More generally, mutations within *gyrA* confer low level fluroquinolone resistance in Gram negative bacteria, while additional mutations in *parC* and *gyrB* are responsible for high level resistance [32]. Ribosomal mutations affecting ribosome assembly are particularly problematic since these lead to large scale transcriptomic and proteomic changes. In *Mycobacterium smegmatis,* such mutations have led to downregulation of KatG catalase (activating enzyme for the drug isoniazid) and upregulation of the transcription factor WhiB7 involved in innate antibiotic resistance. Further, the fitness cost of these mutations is alleviated in a multi-drug environment which promotes the evolution of high-level, target-based resistance [33].

Antiviral resistance is mainly an adaptive process, chiefly driven by mutations [27]. In the case of antiretrovirals used in HIV treatment, the primary mechanism of resistance to most Nucleoside Reverse Transcriptase Inhibitors (NRTI) is through accumulation of mutations near the drug binding site [34]. In Hepatitis B virus, multiple missense point mutations have been linked to several drugs, along with cross resistance observed between drugs [35]. Point mutations in the preS/S region are associated with vaccine failure, immune escape, occult HBV infection and the occurrence of hepatocellular carcinoma (HCC). Similarly, nsSNPS in the preC/C region are related to HBeAg negativity, immune escape, and persistent hepatitis, while those in the X region are implicated in promoting HCC [36]. Likewise, antifungal resistance in *Aspergillus fumigatus* is also primarily driven by mutations in the azole target cyp51A gene [37], while resistance to artemisinin in *P. falciparum* malaria is driven by multiple mutations in the Kelch 13 (K13) propeller protein.

### 1.4 Genomics to identify point mutations linked to AMR

High throughput genomic platforms methods of next generation sequencing (NGS) technologies such as whole genome sequencing (WGS) and genotyping arrays have enabled large scale investigations of AMR for identifying resistance determining genetic variants such as SNPs, INDELs, copy number variation, and frameshift mutations [38], [39], [40], [41], [42], [43]. The role of genetic variants, in particular SNPs, have been implicated in drug resistance by several studies [44], [45], [46], [47]. Building on human complex disease applications [48], [49], [50], genome-wide association studies (GWASs) have been applied to reveal genotype - AMR phenotype associations, at a locus or variant level. Furthermore, GWAS regression models allow the estimation of mutation or genotype effect sizes (e.g. odds ratios). Examples of GWAS analysis in the context of AMR include for *Burkholderia multivorans* [51], *Mtb* [11], [52], [53], severe malaria [50] and fungal pathogens [54].

Bioinformatic approaches exploiting output from WGS technologies and GWAS analyses have enabled AMR prediction and surveillance. Leveraging this wealth of information has enabled novel applications of artificial intelligence and machine learning (AI/ML) in the pan-genome identification of resistance genes, pathways, mechanisms [55], [56], [57], [58], as well as resistance prediction [59], [60], [61]. Bioinformatic approaches have also been used to identify novel drug targets like Inositol-3-phosphate synthase (I3PS) in *Mtb*, opening up new avenues in TB drug discovery [62].

Despite the immense utility provided by genomic analysis, these methods lack the mechanistic underpinning required to develop robust prediction tools [63] necessitating follow-up functional studies [64]. In order to strengthen genomic analysis, it is important to supplement genomic associations with functional consequences of mutations on drug targets. One of the ways to achieve this is via biophysical assessment of mutations on drug-target structure and their interactions.

### 1.5 Biophysical consequences of point mutations on protein structure

The biophysical consequences of protein mutations are mainly studied by assessing thermodynamic stability, which is often used as a proxy for function [65]. This relationship has been clearly demonstrated in the evolution of influenza nucleoprotein which appears to be constrained to avoid low-stability sequences [66]. The synergy between the fields of protein biophysics and protein evolution helps contextualise and rationalise concepts of thermodynamic stability, mutational robustness, evolvability and epistasis in resistance development [67], [68], [69]. Missense mutations resulting in a change in the amino acid may disrupt downstream function by altering protein stability and its associated interactions [70]. For example, three missense point mutations within the *Mtb gidB* gene lead to gidB mutants with lower thermodynamic stability and higher flexibility, considered to be a major driving factor in the emergence of high-level streptomycin resistance [71]. Equally, structural insights into the stability-function relationship have highlighted the rationale for such a trade-off in the development of antibiotic resistance [72].

### 1.6 Using structure to understand impact of point mutations linked to AMR

Structural consequences of point mutations can provide functional insights for resistance phenotypes. For example, point mutations in the Penicillin-Binding Proteins confer resistance to β-lactam antibiotics by making the active site amenable to hydrolysis, or reducing binding affinity for the antibiotic [73]. Structure guided design demonstrated the potential of boronate-based PBP inhibitors to overcome β-lactam resistance in Gram positive organisms [74]. Similarly, missense mutations in the *Mtb gidB* gene (target for the antibiotic streptomycin) are responsible for drug resistance through distortion of the binding pocket affecting SAM (co-factor) binding [71]. Likewise, mutations in *Mtb pncA* gene (target for the pro-drug pyrazinamide) are responsible for the loss of enzyme activity [75]. The underlying mechanism of mutations in the *gidB* gene conferring low and high-level streptomycin resistance in *Mtb* were found to be associated with distortion in the active site morphology by proximal and distal residues affecting the overall structure [76]. Further, the prominent mutation H275Y within the neuraminidase enzyme of the H1N1 pandemic strain renders the drug oseltamivir ineffective due to distortion in the binding pose of the drug within the active site [77]. Structural analysis of C580Y and R539T mutations in the *K13 propeller* gene (associated with artemisinin resistance) in *P. falciparum* malaria revealed local conformational disruption in the mutant and two solvent-exposed patches at conserved sites affecting protein–protein interactions [78].

Structural insights can aid in the absence of phenotypic data [79] as well as provide a physical basis to a more comprehensive understanding of mutational impact on the underlying biological mechanisms. Therefore, computational tools measuring the biophysical effects of resistance linked mutations can aid mechanistic understanding and inform functional studies. Understanding mutational consequences with respect to global (drug-target structure) and local (protein–ligand, protein–protein and protein-nucleic acid) stability effects [80] can be further extended to predict drug resistance for novel mutations [81], [82].

Here, we review several of the principal computational tools and methods currently available for measuring mutational consequences, focusing on those tools which have been used to analyse variation within a pathogen genome and their application in the context of AMR. It is not meant to be an exhaustive list, with other tools available centred on important questions like assessing cancer variations and other human mutations. As such, these go beyond the scope of this review and have been extensively reviewed elsewhere [83], [84], [85].

## 2 Computational tools measuring the effect of mutations

While no general pre-emptive predictor for AMR has been developed, we and others have shown that computational tools for understanding the underlying molecular mechanisms of mutations can be used to identify likely resistant variants [79], [80], [81], [82], [86], [87], [88], [89], [90], [91], [92], [93], [94], [95]. This insight has even been used to guide medicinal chemistry design of inhibitors less prone to resistance [96], [97], [98], [99].

Different tools can be used to describe the effect of mutation on protein function, which may provide an explanation for the AMR phenotype. Some are primarily based on conservation or substitution matrices, and do not require a protein structure as input (Sequence-based methods). Others consider the local environment of the variant within the protein structure in their calculation (Structure-based methods). In the presence of a known AMR-related phenotype, these tools are useful as they provide mechanistic insight which may explain how resistance is brought about at the protein level. Therefore, when analysing specific proteins, it can be beneficial to use different methodologies, as different strategies may give complementary information. Summaries of the types of methods are given below and represent some of the principal tools currently available. Table 1 summarises the main features of some of the currently available tools for analysing effects of pathogen mutations.

**Table1:** Sequence and structure-based tools that predict effect of pathogen missense mutations. The table is an up-to date list of currently available tools (as on 3rd August 2020). The type of method for each tool is specified using the following code; **S**: sequence-based, **St:** structure-based, **SA**: sequence alignment, **SS**: sequence and structure, **(St)**: structure if available. Other abbreviations used: MSA (Multiple Sequence alignment), EC (Evolutionary Conservation), NN (Neural Network), SVM (Support Vector Machine), ML (Machine learning), NMA (Normal Mode Analysis), ΔG: Gibbs free energy in Kcal/mol, ΔΔG: Change in Gibbs free energy in Kcal/mol, wt: wild-type, mt: mutant, K_wt_: affinity of the wild-type protein-ligand complex*, K_mt_:* affinity of the mutant complex*,* RSA: Relative Solvent Accessibility (%).

**Name of tool**	**Type**	**Operating Principle**	**Availability**	**Summary**	**User input**	**Output**	**User Notes**
SIFT: **S**orting **I**ntolerant **F**rom **T**olerant REF: [100]	S	EC	http://sift-dna.org Download: Yes	Calculates a normalised probability of substitution score from multiple alignments based on sequence homology using PSI-BLAST. Removes close homologous sequences to prevent over prediction of “tolerated” substitutions. Mutational effect on protein function is classified as damaging (<=0.05) or tolerated (>0.05).	Fasta sequence or aligned sequences SNP list	Per-SNP: 1) SIFT score 2) Binary mutation classification 3) Median sequence conservation	Predictions for submitted SNPs, as well as all possible SNPs (but without a score). Positions are weighted equally within an alignment. Alignments may be user defined. Sequence conservation score provides a useful estimate of whether the alignment contains sufficient variation to support classification.
PROVEAN: **Pro**tein **V**ariation **E**ffect **An**alyzer REF: [104]	S	EC	http://provean.jcvi.org/seq_submit.php Download: Yes	Related sequences are collected with BLAST (using CD-HIT) and clustered based on 75% global sequence identity. The top 30 clusters of closely related sequences form the supporting sequence set, used to generate the prediction. Delta alignment scores are computed for each supporting sequence and averaged within and across clusters to generate the final PROVEAN score. Predicted mutation effects are classified as either deleterious or neutral based on a predefined threshold (-2.5). Available as: PROVEAN Protein PROVEAN Protein Batch* PROVEAN Genome Variants* *Human and Mouse only	Fasta sequence Mutation list (SNPs and INDELs)	Per-mutation: 1) PROVEAN score 2) Binary mutation classification	Predictions for submitted mutations only. Predict effects for both SNPs and INDELs, but not frameshift mutations. Batch processing of multiple organisms. The classification threshold is fixed in the online version. Stand-alone package only available for PROVEAN Protein.
SNAP2: **S**creening for **N**on-**A**cceptable **P**olymorphisms, v2 REF: [105]	S	NN	https://www.rostlab.org/services/SNAP/ Download: Yes	Combines evolutionary information with an expanded list of original SNAP features (amino acid properties) including features such as AA index, predicted binding residues and disordered regions, residue annotations from Pfam and PROSITE, etc. Mutations are classified as either neutral or effect based on predicted scores, between (-100 to 100) respectively. The prediction algorithm is based on a NN consisting of a feed-forward multi-layer perceptron. 10-fold cross-validation is used to create 10 models, each providing a single score for each output class (neutral/effect). The final score is calculated as the difference between the average scores for each output class.	Fasta sequence	For all possible substitutions: 1) Heatmap representing the predicted effect 2) Multi column table with Predicted Effect, Score and Accuracy.	Predictions for all possible substitutions. Prediction scores are accompanied by an “accuracy metric” to aid interpretation. Uses predicted structural features. Heatmap generated for visualisation of the predictions. Additional method (SNAP2noali) predicts functional effects without alignments. Automatic selection of best method (SNAP2 by default, and SNAP2noali for orphans) with notification to users.
ConSurf REF: [106]	S(St)	EC	https://consurf.tau.ac.il/ Download: No	Estimates evolutionary conservation rate of amino/nucleic acid positions based on the phylogenetic relations between homologous sequences. Homologous sequences are searched using CSI-BLAST, PSI-BLAST or BLAST, with closely related sequences removed using CD-HIT with multiple sequence alignments (MSA) generated by MAFFT by default. MSA is used to construct phylogenetic relationships using the neighbour joining method. Position specific evolutionary rates are calculated using the empirical Bayesian or Maximum Likelihood methods. Scores graded 1 (variable) to 9 (conserved) for visualisation.	Amino acid/nucleotide sequence Structure (if available) MSA (if available) *Advanced options:* - homologue database - MSA methods - Phylogenetic tree - structural data - calculation method - evolutionary substitution model Optional: user defined MSA and phylogenetic tree.	Detailed output containing conservation scores, MSA and BLAST results. Estimates mapped onto sequence and structure.	Analysis at amino acid and nucleotide levels. Improved HMMER algorithm to search for homologous proteins. Results are accompanied by confidence intervals. Robust statistical approach to differentiate between apparent conservation (short evolutionary time) and genuine conservation (purifying selection). ‘ConSeq’ mode used in the absence of a structure. Site-specific predictions of the buried/exposed status of each position.
MAPP: **M**ultivariate **A**nalysis of **P**rotein **P**olymorphism REF: [110]	SA	EC	Download only: http://mendel.stanford.edu/SidowLab/downloads/MAPP/index.html	Combines MSA with 6 physicochemical properties for amino acids. Calculates a MAPP impact score for each position within the MSA. Sequences in the MSA are weighted to account for phylogenetic correlation. Physicochemical property scores for each column along with their mean and variances are calculated. The deviation of each property is calculated for every possible variant and converted to a single score.	Fasta format MSA Phylogenetic tree	Multicolumn table giving the physico-chemical characteristics of each position, MAPP impact score, and a listing of “good” and “bad” amino acids.	Predictions for every possible substitution, and median MAPP scores calculated for each position. Constructs a physiochemical profile rather than an amino acid profile. Demonstrates value of using only orthologous protein in creating a conservation profile. Scores are continuous and interpreted in a relative manner with higher MAPP scores indicating more conserved areas. Can be optimised for individual genes including MAPP impact score threshold for classification. Requires user defined MSA and phylogenetic tree.
PANTHER-PSEP: **P**rotein **AN**alysis **Th**rough **E**volutionary **R**elationships-**P**osition **S**pecific **E**volutionary **P**reservation REF: [149]	S	EC	http://www.pantherdb.org/tools/csnpScoreForm.jsp Download: Yes	Uses Hidden Markov Model (HMM) to align sequence to protein families and subfamilies in its database to calculate the evolutionary preservation metric. Uses variation over each alignment position to estimate the likelihood of a coding SNP to cause a functional impact on the protein. Score represents the time (in millions of years [my]) a given amino acid has been preserved in the lineage, directly corresponding to the likelihood of a functional impact. Score classified into: Probably damaging, Possibly damaging, Probably benign.	Fasta sequence SNP list *Other parameters:* Organism	Per-SNP: 1) Preservation Time: PANTHER PSEP score 2) Message: Classification of the PSEP score	Positions are weighted equally at all positions within an alignment. Profiles are subfamily specific if they substantially differ from entire family. User defined alignments are not possible since scores are derived from HMMs (PANTHER protein library) together with an ontology of protein function (PANTHER/X – a simplified form of GO) to make predictions.
FoldX suite REF: [113]	St	Empirical force field	Download only: http://foldxsuite.crg.eu/	Empirical force-field used for calculating mutational effects of stability, folding, and dynamics on proteins and nucleic acids ΔG (free energy of unfolding) is calculated using a combination of empirical terms. Empirical data (derived from protein engineering experiments) is used for weighting energy terms for stability calculations. Foldx *BuildModel* command calculates stability changes upon mutation based on a full atomic description of the protein structure. Classification of ΔΔG: ΔΔG > 0: Destabilising ΔΔG < 0: Stabilising	PDB file SNP list (including chain ID)	Multiple output files where requested. Main output is present in ‘*Dif_’’* files, containing ΔG of wt and mt residues along with ΔΔG of mutation. Output also contains changes in the associated energy terms.	Command line interface. Creates mutant structure models. Can be used to analyse protein–protein and protein-DNA interactions. Calculates actual stabilities of wt and mt structures, as well as change in stability upon mutation (ΔΔG). Easily integrated into custom workflows. Optimised energy function for faster calculations. Requires registration to download.
PoPMuSic (v2.1): **P**rediction **O**f **P**roteins **MU**tations **S**tab**I**lity **C**hanges REF: [115]	St	Physics-based and NN	https://soft.dezyme.com/ Download: No	Stability change upon mutation calculated using a linear combination of statistical energy potentials, accounting for variation in volume of the mutant residue. Predictive models include an optimised set of 52 parameters, whose values are estimated and optimised using a neural network. ΔΔG of point mutation is calculated by a linear combination of 16 terms: 13 statistical potentials, 2 terms for volume of wt and mut residues, and 1 independent term. Classification of ΔΔG: ΔΔG > 0: Destabilising ΔΔG < 0: Stabilising Additional “optimality” score is assigned for each position in the protein sequence. It indicates poorly optimised positions with potential functional consequences.	Only accepts currently available entries in the PDB SNP list in three input modes: 1) Systematic: all possible point mutations 2) Manual: single mutation 3) File: SNP list	Multi-column table containing secondary structure, solvent accessibility (%) and predicted ΔΔG of mutations.	Optimised to rapidly calculate stability changes of all possible mutations in mid-size proteins. Graphical output of sequence optimality scores. No option to upload user-defined PDB files. Requires registration to download.
I-Mutant (2.0) REF: [116]	S(St)	SVM	http://gpcr2.biocomp.unibo.it/cgi/predictors/I-Mutant3.0/I-Mutant3.0.cgi Download: No	Predicts stability effect of a point mutation, as a classification or regression task. The classification task predicts the direction of change, while the regression estimator predicts the ΔΔ*G*. Can be applied to both sequence and structure. RI value (Reliability Index) is computed from the output of the SVM model. Binary classification ΔΔG: ΔΔG < 0: Decrease Stability ΔΔG > 0: Increase Stability Ternary Classification ΔΔG: Large Decrease of Stability: ΔΔG < -0.5 Large Increase of Stability: ΔΔG > 0.5 Neutral Stability: 0.5<=ΔΔG<=0.5	Fasta sequence or PDB code/file Chain ID Single SNP Temperature PH Prediction request: Binary/Ternary classification	Table containing: 1) RSA (%) of mt residue 2) RI (Reliability Index) 3) Predicted ΔΔG 3) Classification of ΔΔG	Predicts both the direction and the estimate of stability. Experimental conditions of pH and Temperature (Celsius) are considered in the stability calculations. Analyses a single mutation at a time only. Output on the web server is better than output requested via email. Use of two different SVM models can lead to discordance between the ΔΔ*G* sign and classification, but is stated to occur only in cases of low RI value.
STRUM: **STR**ucture-based prediction of protein stability changes **U**pon single-point **M**utation REF: [117]	S(St)	ML	https://zhanglab.ccmb.med.umich.edu/STRUM/ Download: Yes	Calculates ΔΔG of mutation using gradient boosting regression algorithm trained on 120 features divided into three groups (sequence, threading and structure). Classification of ΔΔG: ΔΔG < 0: Destabilising ΔΔG > 0: Stabilising	Fasta sequence or PDB file SNP list in two modes: 1) Single or multiple SNPs 2) Systematic: All possible SNPs for user defined amino acid segments.	Results available via e-mail only. Multi-column table containing ΔΔG for SNPs.	Combines sequence profiles and 3D features 3D Structure modelling of query protein sequence by iterative threading assembly refinement simulations Computationally expensive with relatively long runtime.
MAESTRO: **M**ulti **A**g**E**nt **ST**ability p**R**edicti**O**n REF: [150]	St	Multi agent: ML methods and statistical scoring functions	https://pbwww.che.sbg.ac.at/maestro/web Download: Yes	Multi-agent method where 3 ML methods i.e Artificial NN, SVM and Multiple Linear Regression. are combined to generate a consensus prediction. Each agent (ML method) uses 9 input values divided into two categories: SSF functions and protein properties (size, mutational environment, etc.). Classification of ΔΔG: ΔΔG > 0: Destabilising ΔΔG < 0: Stabilising	PDB code/file Input mode: 1) Specific mutations 2) Sensitivity profile: all possible mutations 3) Scan for destabilising mutations 4) Stability of Disulphide bonds	Input modes 1 & 2 ΔΔG predictions and confidence intervals. Graphical display.	Ability to analyse mutations independently or in combination ΔΔG predictions are accompanied by confidence intervals. High throughput scanning for all possible point mutations. Specific mode for prediction of stabilising disulphide bonds.
mCSM suite: **m**utational **C**ut-Off **S**canning **M**atrix REF: [122]	St	Graph-based and ML	Protein Stability (PS), Protein-Protein (PP), Protein-DNA (P-NA) http://biosig.unimelb.edu.au/mcsm/ Download: No	Uses graph-based methods to calculate atomic pairwise distance surrounding the wt amino acid. Mutational impact is captured based on a change in the atomic pharmocophore count resulting from the point mutation. Together, this forms the mCSM-signature, and is used to train predictive models for analysing mutational impact on structure stability. Predicted ΔΔG < 0 relates to destabilising, and ΔΔG > 0 relates to stabilising mutational effects. Ternary Classification of Destabilising effect: Mild: −1 < ΔΔG < 0 Moderate: −2 < ΔΔG < -1 High: ΔΔG < -2 Ternary Classification of Stabilising effect: Mild: 0 < ΔΔG < 1 Moderate: 1 < ΔΔG < 2 High: ΔΔG > 2	PDB code/file SNP list Chain ID Input modes: 1) Single mutation 2) Mutation list 3) Systematic: all possible mutation for a single residue	Input mode 1: 1) Predicted ΔΔG 2) Classification of mutational stability change Input modes (2) & (3): Multi-column table with predicted ΔΔG and RSA.	Predicts both the direction and the estimate of stability. Mutant structure is not required. webGL structural visualisation for input mode 1. Works at an atomic level. Demonstrates correlation between atomic-distance pattern of the wild-type residue environment and mutational impact. Calculates overall stability of protein and interactions.
mCSM-lig: **m**utational **C**ut-Off **S**canning **M**atrix on **lig**and affinity REF**:**[88]	St	Graph-based and ML	Protein-ligand affinity (mCSM-lig): http://biosig.unimelb.edu.au/mcsm_lig/prediction Download: No	Based on the mCSM graph-based signatures (as above) with the addition of small-molecule chemical features and ligand physicochemical properties to capture mutational changes. Predictive models trained on a representative set of protein–ligand complexes. Mutational impact on affinity is calculated as the log (ln) affinity fold change as below: ln(K_wt_) - ln(K_mt_) = ln (fold-change) Classification of ln (fold-change): ln (fold-change) < 0: Destabilising ln (fold-change) > 0: Stabilising	PDB code/file Single SNP Chain ID 3-letter ligand ID wt-affinity (nano Molar (nM))	Log affinity fold change Distance to ligand (Angstroms) DUET stability change (Kcal/mol) Binary classification of affinity and stability changes.	Predicts both the direction and the estimate of stability. Returns both DUET and ligand affinity changes, along with ligand distance to site. Measures both global and local stability effects. Analyses single mutation at a time. Returns a change in affinity value. Less reliable results for sites > 10 Å from ligand.
Rosetta Flex_ddG REF**:**[151]	St	All-atom energy function	Download only: https://www.rosettacommons.org/software/license-and-download	Based on a mixed physics and knowledge-based approach. Uses all-atom energy function, parameterized from small molecule and X-ray crystal structure. The *Flex_ddG* protocol models changes in the ΔΔG upon mutation at a protein–protein or protein–ligand interface using the ‘backrub’ algorithm. This algorithm is used to sample conformational space and produce an ensemble of wt and mt models to estimate the interface ΔΔG values. Ternary Classification of ΔΔG: Destabilising: ΔΔG >=1 Neutral: −1 < ΔΔG < 1 Stabilising: ΔΔG<= −1	Customized PDB file Ligand parameter file Customized XML protocol file Mutation list Chain ID	Each run outputs db3 file containing the changes in the main components of the energy function, ΔG wt, ΔG mt, and the ΔΔG upon mutation.	For a reliable prediction, at least 35 runs per mutation are required, with each run taking between 2 and 4 h. Access to HPC may be required for large number of mutations. Protocols are written in XML format. Requires license to download.
INPS-MD **I**mpact of **N**on-synonymous mutations on **P**rotein **S**tability-**M**ulti **D**imension REF: [141]	S/St	SVM regression	https://inpsmd.biocomp.unibo.it/inpsSuite Download: No	Calculates ΔΔG of mutation on sequence and structure. The sequence-based predictions are derived from seven descriptors to account for evolutionary information (INPS), while two additional structural features (RSA and energy difference between wt and mt structures) are included for the structure-based predictions (INPS-3D). SVM regression is used to map the sequence descriptors to the ΔΔG values. Classification of ΔΔG: ΔΔG < 0: Destabilising ΔΔG > 0: Stabilising	Fasta sequence/PDB file SNP list Chain ID (INPS-3D only)	Per SNP in list: Predicted ΔΔG	Predicts both the direction and the estimate of stability. Can operate on both sequence (INPS) and structure (INPS-3D) Accounts for anti-symmetric property of variation i.e ΔΔG (A->B) = - ΔΔG (B->A).
DeepDDG/ iDeepDDG REF: [142]	St	NN/ Ensemble method	http://protein.org.cn/ddg.html Download: No	Calculates ΔΔG of mutation using NN trained on nine categories of sequence and structural features. Operates independently as ‘DeepDDG’, and in an integrated manner as ‘iDeepDDG*’.* In the latter, predictions from three methods: mCSM, SDM and DUET are fed into the concatenation layer of the NN to generate the consensus prediction. Classification of ΔΔG: ΔΔG < 0: Destabilising ΔΔG > 0: Stabilising	PDB code/file Network model: -DeepDDG -iDeepDDG SNP list in two modes: 1) Single or multiple SNPs 2) All possible mutations	Per SNP/all possible SNPs: Predicted ΔΔG	Predicts both the direction and the estimate of stability. Accounts for anti-symmetric property of variation i.e ΔΔG (A->B) = - ΔΔG (B->A). Runs in independent or integrated modes. ‘DeepDDG’ allows high throughput scanning for all possible point mutations with relatively fast computation time. For running ‘iDeepDDG’, user must provide predictions for each mutation from the mCSM DUET server.
DUET REF: [102]	St	Ensemble method: SVM	http://biosig.unimelb.edu.au/duet/ Download: No	Predicts stability effects upon mutation on proteins. Combines predictions from two complementary methods: mCSM and Site Directed Mutator (SDM) in an optimised predictor to generate the DUET prediction. The optimised predictor is generated using SVM trained with Sequential Minimal Optimisation. Classification of ΔΔG: ΔΔG < 0: Destabilising ΔΔG > 0: Stabilising	PDB code/file SNP list Chain ID Input mode1: Single mutation Input mode 2: Systematic: all possible mutation for a single residue	Input mode 1: 1) Predicted ΔΔG from mCSM, SDM and DUET. Input mode 2: Multi-column table with predicted ΔΔG from mCSM, SDM, DUET and RSA.	Predicts both the direction and the estimate of stability. Mutant structure is not required. webGL structural visualisation for input mode 1.
ELASPIC: **E**nsemble **L**earning **A**pproach for **S**tability **P**rediction of **I**nterface and **C**ore mutations REF**:**[124]	(St)	Ensemble method: ML	http://elaspic.kimlab.org/ Download: No	Predicts stability effects upon mutation in both, domain cores and domain-domain interfaces. Combination of semi-empirical energy terms, sequence conservation, and a wide variety of molecular details with a Stochastic Gradient Boosting of Decision Trees (SGB-DT) algorithm. Uses a combination of sequence, molecular and energy features including prediction scores from other tools.	Uniprot Protein ID or PDB structure SNP list	Multi-column table, with the main output being ΔG of wt and mt structures, and ΔΔG of mutation. Results are downloadable. FoldX generated mutant structures in pdb format Jmol applet showing superimposed wt mt structures.	Can be run as a single or multiple mutations and Protein-protein interactions Option to filter results based on additional criteria. Non-human proteins may take longer to run. An interactive connectivity network showing the affected protein–protein interaction mutations.
DynaMut REF**:**[118]	St	Ensemble method: NMA	http://biosig.unimelb.edu.au/dynamut/ Download: No	Predicts stability effects based on protein dynamics resulting from vibrational entropy changes. Integrates mCSM signatures and normal model analysis. Combines mutational effect from 3 structure-based prediction tools to generate a consensus prediction. Classification of ΔΔG: ΔΔG < 0 Destabilising ΔΔG > 0: Stabilising	PDB code/file Single SNP/SNP list Chain ID	NMA based predictions Other structure-based predictions included.	Accounts for protein molecular motion and flexibility. Easy and detailed visualisation of results including interatomic interactions, deformation and fluctuation analysis. Returns a change in stability. Computationally expensive with relatively long runtime.

### 2.1 Sequence-based methods

As these methods rely solely on the gene or protein sequence, they are often useful in the absence of a known protein structure or when homology modelling is not possible. The predictions from these tools are generally based on sequence alignments, predicted secondary structures and subsequent conservational trends. Most methods determine a score with cut-offs leading to functional classification of mutations into deleterious or neutral. This functional classification is not always applicable to AMR mutations, as variants may be ‘deleterious’ to protein conservation, but gain-of-function through survival in the presence of drug. For example, when analysing rifampicin resistant *Mtb* mutations we found that they tended to cluster within more conserved regions of the *rpoB* gene [80] (Portelli and Ascher, personal communication). Similar analysis carried out on pyrazinamide [82] and bedaquiline [81], revealed that known resistant *Mtb* mutations were more likely to lead to deleterious effects compared to susceptible variants in the same gene [100]. However, when measuring mutational tolerance [101], strong evidence of positive selection for resistant mutations was observed. Therefore, the utility of these tools in understanding AMR mechanisms lies in the actual scores, where a comparison of different scores across variants, accounting for their genetic position can uncover important underlying mechanisms and trends related to evolutionary conservation. We have previously shown that this sequence information is also complementary to structural information, particularly within the context of machine learning [102]. Several of the major methods which are applicable across pathogens and human genomes are:

#### a SIFT

The SIFT (Sorting Intolerant From Tolerant) can be used to analyse missense mutations and INDELs. The SIFT scoring method combines sequence alignment with a position-specific scoring matrix (PSSM), which accounts for the likelihood of an amino acid to occur within a specific position. The amino acid chemical properties are also incorporated to determine a scaled probability of the mutation (SIFT score), on which the output (tolerated or deleterious) is based [100]. SIFT has been used to build the Variant Effect Predictor (VEP) tool developed as part of the Ensembl 2018 project [103].

#### b PROVEAN

PROVEAN (Protein Variant Effect Analyzer) is able to account for (multiple) missense mutations and INDELs. It uses the BLOSUM62 substitution matrix as an amino acid probability matrix and combines this with differences in sequence similarity between wild-type and mutant sequences. The sequence context in which variation occurs is also considered, to represent environmental surroundings and effects. A numerical score is generated for each variant, which enables the functional classification into deleterious or neutral [104]. PROVEAN scores have provided the evolutionary basis for the recently deployed web-based tool SUSPECT-PZA [82] which predicts pyrazinamide (PZA) resistance mutations in the *Mtb pncA* gene.

#### c SNAP2

SNAP2 (Screening for Non-Acceptable Polymorphisms v.2) characterises the effect of all possible missense mutations as either neutral or deleterious. It is a machine learning-based predictor trained on neural networks. It also accounts for amino acid position probabilities using position-specific independent counts, based on the BLOSUM62 matrix. This predictor considers other features such as protein fold (Pfam, PROSITE) and functional annotations (SWISS-PROT) during training, and as such is the tool that spans the most comprehensive feature space [105]. As well as forming part of the SUSPECT-PZA tool [82], SNAP2 scores have provided the evolutionary basis for a similar tool called SUSPECT-BDQ [81]. This tool predicts the effect of missense mutations on the anti-TB drug bedaquiline, reserved to treat MDR and XDR TB.

#### d ConSurf

ConSurf estimates an evolutionary rate score for every position across the sequence, unlike the tools above which base functional classification on score thresholds. In the context of drug resistance, it can help identify sites which are likely to lead to resistance if mutated. The ConSurf score is based on a multiple sequence alignment, which generates probabilistic evolutionary models and phylogenetic links. Through this score, more conserved sites (having slower evolutionary rates), which have important functional and structural consequences are identified [106]. Consurf has been used to estimate and visualise conserved regions within SARS-CoV-2 [107], the SARS-CoV nsp12 polymerase domain [108], and the S2 subunit in MERS-CoV isolates [109] to aid antiviral strategies.

#### e Mapp

MAPP (Multivariate Analysis of Protein Polymorphism) predicts the functional impact of all possible missense mutations. It combines evolutionary conservation and physicochemical information. It uses data from multiple sequence alignments from orthologs to estimate a mean for each of the six physicochemical properties (hydropathy, polarity, charge, volume, and free energy in alpha helices and beta strands) for each position. A single composite value for each physicochemical value is generated based on the deviation from the mean for all twenty amino acids. High MAPP scores indicate highly conserved sites, which in the context of drug resistance can indicate resistance promoting sites [110]. MAPP has been used to develop the ProPhylER [111] tool, used for proteome wide investigation of mutational impact on eukaryotic protein.

### 2.2 Structure-based methods

When analysing missense mutations, structure-based methods can offer a 3-dimensional explanation of molecular consequences of mutations, which may not be evident from sequence analysis alone [86], [89]. These methods include the analysis of the protein structural and functional consequences of mutations, including those on protein folding, stability, dynamics, and alterations to interactions with normal ligands. Protein structure information can be incorporated through rule-based or machine learning based approaches (see Table 1). As acquired resistance can develop through missense mutations, analysing their effects can inform on underlying mechanisms of resistance. In previous analyses, we observed that known resistance mutations arising in the drug-target tend to significantly reduce functional affinities, such as nucleic acid affinity [80], [81], [82], [93], [94], [95]. Resistance mutations in drug activators are associated with large decreases in protein stability or activity [79], [80], and those in drug exporters tend to increase protein flexibility to promote drug export [91]. To run these predictors, a crystal structure of the protein or a homology model is required. A summary of the principle methods and applications are described below:

#### 2.2.1 Measures of protein stability

The introduction of resistance-causing missense mutations to a protein structure rarely comes at a negligible cost to protein stability, whether decreasing local stability and affecting protein folding, or increasing local stability and compromising wild-type protein dynamics [112]. Therefore, quantifying the effect of missense mutations on stability presents a good starting point in understanding the basic variant protein changes. Computational tools predicting thermodynamic stability of a protein do so by estimating the Gibbs free energy (ΔG Kcal/mol). The subsequent impact of a point mutation on protein stability is then estimated as a change in the Gibbs free energy (ΔΔG Kcal/mol) between wild-type and mutant proteins, or vice versa. Additionally, these tools provide both the extent (the actual value of ΔΔG) as well as the direction (destabilising/stabilising) of the resulting mutational effect. Different *in silico* protein stability predictors are available, of which we highlight a few, based on the methodologies considered in their approximations. Further details for these (and additional) methods can be found in Table 1**.**

a. FoldX is an empirical-based predictor which provides information on how a single point mutation alters the stability of a protein. It constructs structure models of the protein with the mutation and estimates the stability (ΔG) associated with the mutant protein. Estimation of stability is based on intramolecular interactions such as van der Waals’ forces, solvation energies, interactions with water, hydrogen bonds, electrostatic effects and main and side chain entropies. Mutational impact is calculated through a weighted summation of all the intramolecular interactions, and estimated as a change in stability (ΔΔG) between mutant and wild-type structures. In this way, ΔG for each mutant protein, ΔΔG upon mutation, and the contribution of each intramolecular interaction, are made available to the user. The extent of the mutational impact (the value of ΔΔG) as well as the direction of change (ΔΔG < 0: stabilising, ΔΔG > 0: destabilising) are captured by the predictions [113], [114].
b. PoPMuSic (v2.1) is a statistical method which uses knowledge-based potentials to predict mutational impact on the stability of a protein. It returns the predicted ΔΔG of a single point mutation of a protein and is able to systematically analyse this for all possible point mutations for a given protein. Additionally, an ‘optimality’ score for each amino acid in the sequence with respect to stability is returned. The optimality score identifies sites of structural weakness i.e. clusters of residues that are considered non-optimal from an evolutionary perspective. Therefore, mutations with desired stability properties (ΔΔG < 0: stabilising, ΔΔG > 0: destabilising) and poorly optimised positions can be identified. These sites can relate to the protein’s function, and be used for rational protein design and other experimental studies. In PoPMuSic, a protein is represented as a statistical potential based on individual residue properties such as sequence position, conformation, solvent accessibility, or a combination of inter-residue distances. The optimality score is computed from the sum of the predicted ΔΔG of all stabilising mutations at a given position in the sequence. Since the majority of the mutations have a destabilising effect, this score is expected to be close to zero for most positions in the sequence, with high negative values indicating sites with strongly stabilising mutations and/or several stabilising mutations with mild effect [115].
c. I-Mutant (v2.0) is an ML based predictor which computes mutational stability changes using support vector machines. It provides an estimate of the ΔΔG upon a single point mutation based on protein structure (or sequence). The resulting ΔΔG highlights the extent as well as the direction of impact (ΔΔG < 0: destabilising, ΔΔG > 0: stabilising) on the protomer. The predictions consider the mutated residue environment as a 9 Å region (structure) and a 19-residue window (sequence) surrounding the mutation. This environment is combined with experimental pH and temperature conditions, enabling the user to define different pH and temperature conditions on a case-by-case basis to better encompass protein biological conditions [116].
d. STRUM is an ML based predictor and returns an estimate of the ΔΔG of a single point mutation on 3D models based on wild-type sequences. It can be used to analyse single mutations or all possible mutations within a specified region of the protein. Similar to methods above, both the magnitude of change as well as the direction (ΔΔG < 0: destabilising, ΔΔG > 0: stabilising) are encapsulated in the predictions. The 3D models are generated using iterative threading assembly and combined physics- and knowledge-based energy functions. Predictors are trained based on 3 groups of features: sequence, threading, and I-TASSER structure. A total of 120 features are trained through Gradient Boosted Regression Trees (GBRT) to overcome overfitting effects [117].

#### 2.2.2 Measures of global and local stability within a single framework

**Fig. 1:**
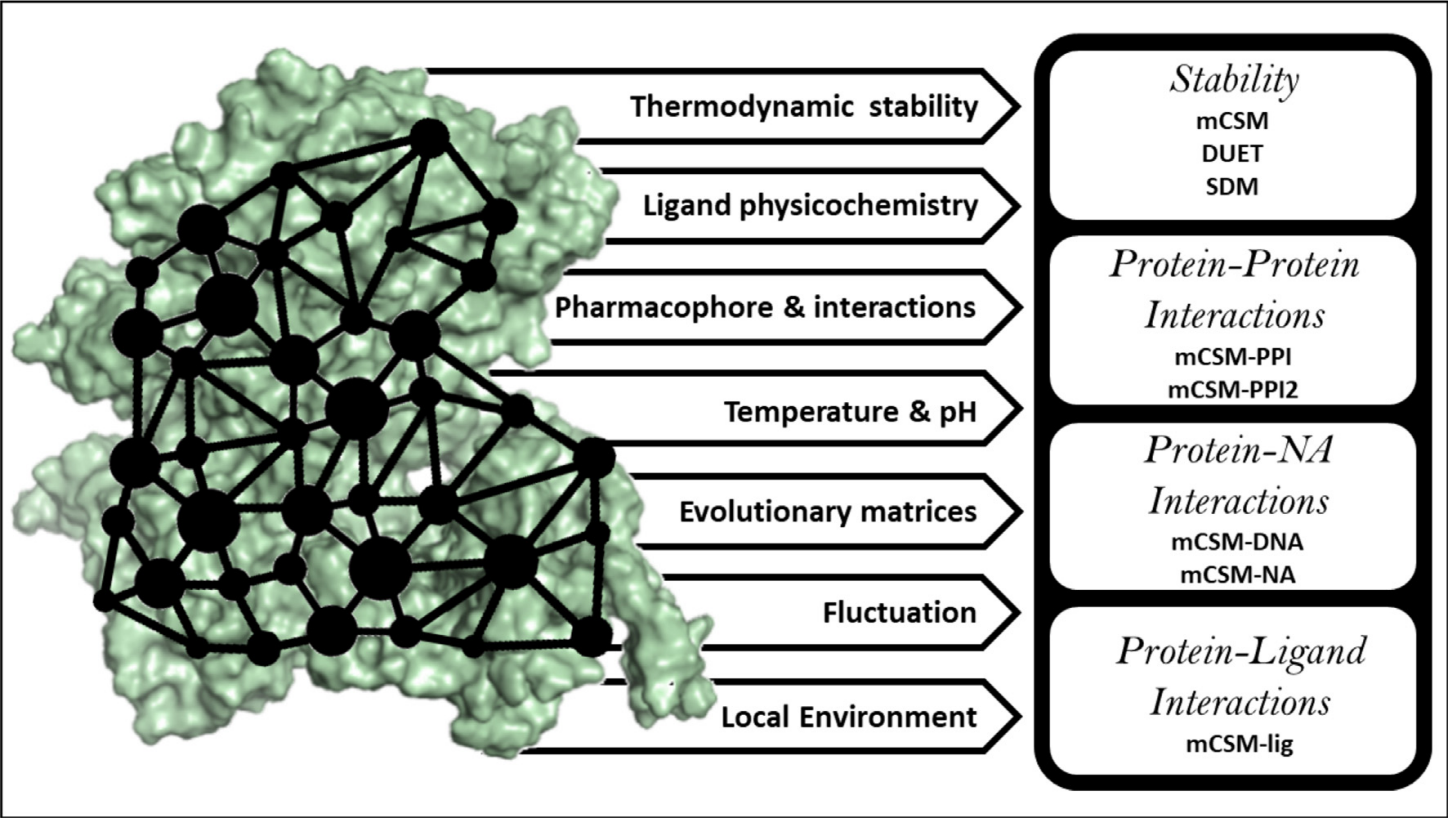
A summary of mutational Cut-off Scanning Matrix (mCSM) method and its application in measuring mutational effects on protein stability (mCSM DUET), protein–protein interaction (mCSM-PPI, mCSM-PPI2), protein-nucleic acid (mCSM-NA) and protein–ligand affinity (mCSM-lig).

The mCSM (mutation Cut-off Scanning Matrix) suite of computational tools accounts for the changes in protein stability dynamics [118], and interactions with other proteins [119], ligands [88] and nucleic acids [120] upon introduction of missense mutation. It estimates change in stability (ΔΔG) and change in binding affinity of the ligand. Measuring the impact of missense mutations beyond protein stability, by looking at functional affinities, is crucial to characterise the mechanisms of AMR-associated mutations. This is because affinities to ligands, nucleic acids and other proteins are highly dependent on specific interaction sites, irrespective of protein stability changes. Functionally, protein affinity changes to its ligand is especially important in AMR, as it enables the identification of mutations directly affecting ligand binding. The extent of this importance, however, relates to the drug mode of action, meaning that other functional affinities should also be considered to identify mechanisms beyond direct ligand binding. The mCSM suite of tools quantify these stability and functional measurements using graph-based signatures [121], which summarise the global environment of the protein as a series of nodes for each atom, and represents the local environment at the mutation site as edges on the graph between the nodes at similar distances from the mutation. A pharmacophore count is appended to these signatures to account for any physicochemical changes imparted by the missense mutations [122] **(**Fig. 1**).** Through this graph-based network, the impact of a missense mutation over the whole protein can be calculated. All methods within the mCSM suite are based on ML approaches in quantifying missense mutational changes, and are freely available via their respective web servers.

Ensemble methods like DUET [102] generate a consensus prediction based on two different tools, while the *meta*-predictor tool by Broom, et al. [123] combines predictions from eleven available tools. Similarly, the ELASPIC method [124] combines semi-empirical energy terms, sequence conservation, and several molecular features to predict mutational effect on stability and affinity. Likewise, DynaMut [118] combines graph-based structural predictions with Normal Mode Analysis to account for protein dynamics and molecular motion to assess mutational impact. Consensus approaches have the advantage of improved accuracy over individual tools, but are tightly coupled and sensitive to their availability.

#### 2.2.3 Insights from molecular dynamics simulation experiments

Despite not providing direct thermodynamic measures of mutations, molecular dynamics (MD) remains an invaluable technique for analysing mutational effects on protein conformational movement, especially considering that other techniques run on static protein structures. In the context of AMR, MD simulations enable comparison between wild-type and mutant protein trajectories. Visualising these differences can highlight co-occurring mutations and sites with local protein rigidification. Different MD techniques may be used, depending on computational cost and the level of throughput required.

An all-atom MD method has been adopted to study co-occurring missense mutations V82F/I84V (known to confer resistance to target inhibitors) within HIV-1 protease [125]. This analysis enabled the characterisation of an equilibrium shift imparted by these mutations from a closed to a semi-open conformation as a possible cause of drug resistance [125]. More recently, the effect of G140S mutation on HIV-1C Integrase (IN) protein provided insight into dolutegravir resistance. Decreased stability of IN and higher flexibility around the 140 loop region in the mutant system reduced drug affinity [126]. Similarly, MD simulations also examined artemisinin resistance in malaria. Mutation R539T and C580Y in the *P. falciparum* K13 region revealed local structural destabilisation of the Kelch-repeat propeller (KREP) domain but not the overlapping shallow pocket [78]. In fungal and bacterial enzymes, MD investigation of the interaction of triazole drugs with their target, CYP51, has highlighted the potential to design inhibitors with greater ortholog specificity. While protein-fluconazole interactions were strongly mediated by ligand-HEME interactions in fungal enzymes, the same was mediated by polar interactions in the bacterial counterpart (CYP51 *Mtb*) [127]. Stereochemical changes, rather than electrostatic effects, of ten point mutations in *Mtb katG* led to isoniazid (INH) resistance by restricting access of the drug to its catalytic site [128]. Likewise, conserved motions and unbinding events of 82 point mutations in *Mtb pncA,* linked to PZA resistance, were also discerned through MD simulations. Coupled expansions and contractions of the pncA lid and the side flap were observed in the unbinding of PZA in some mutants, while destabilisation of the “hinge” or nearby residues facilitated lid opening and PZA release from the active site [129].

MD studies have also shed light on AMR mutations in biological pathways. For example, mutations Y59H, M84I and E160D within the RamR homodimerization domain on *ramA* promoter were shown to affect structure stability and binding affinity. These mutations led to dysregulation of the multidrug efflux pump RND, and consequent drug resistance in *Salmonella enterica* [130]. Another example, where extensive modifications modelled by MD simulations of six missense mutations in Thymidylate synthase A (ThyA), a key enzyme in the *Mtb* folate pathway, provided a deeper understanding of Para-aminosalicylic acid resistance [131]. Likewise, investigation of inhA-INH resistance in *Mtb* revealed a ligand “locking” mechanism together with increased vibrational coupling between inhA cofactor binding site residues, responsible for the inhibitory function of the wild-type complex. This insight provided an explanation of how the resistant mutation S94A circumvents these subtle changes in global structural dynamics, with downstream effects in the fatty acid synthase pathway [132]. All-atom MD simulations have also been used to understand the mechanism of anti-microbial peptides within biofilms, which can potentially serve as alternative therapies in the presence of AMR [133].

Although, an all-atom MD approach offers detailed analysis of specific mutations, it is often computationally expensive making it impractical for large mutational datasets. In such cases, an approximated MD technique, known as normal mode analysis (NMA) can be adopted. NMA uses harmonic motion to summarise protein dynamics arising from vibrational entropy changes. This approach is the basis for DynaMut [118] (part of the mCSM-suite of computational tools described above) which predicts missense mutational impact on proteins while accounting for their molecular motions.

## 3 Applications of the computational tools for characterising drug resistance in TB and other infectious diseases

The tools described above for measuring the effects of mutations within a gene have been used to provide a molecular understanding of how variants can affect pathogen drug resistance in *Mtb* [80], [92] and *P. vivax* [134]. In all cases, the different tools have provided complementary information to describe mutational effects under selective pressure as a balance of fitness costs across different protein properties.

To demonstrate the utility of this approach, we explore in more detail *Mtb* variants in two genes *katG* (resistance to isoniazid) and *rpoB* (resistance to rifampicin), which have been associated with drug resistance from GWAS analyses [11], [45]. Most *katG* mutations conferred resistance through a disruption of protein stability [80]. Functionally, it is thought that *Mtb* renders the non-essential KatG unstable to impede the activation for prodrug isoniazid, thereby conferring resistance. When considering rifampicin resistant mutations within gene *rpoB*, we found that most mutations disrupt protein–protein interactions, leading to a loss in nucleic acid affinity. Structurally, the effects of these mutations within RpoB, the β-subunit of RNA polymerase, are compensated for by mutations within RpoC, which is the β′ subunit, thereby restoring normal functioning of the RNA polymerase, with an added resistance property [135], [136], [137].

Within this analysis, two distinct classes of mutations were observed: (i) those having high allele frequency within GWAS, but which had mild overall effects on protein stability and affinities to ligands, other proteins and nucleic acids, and (ii) those having lower allele frequency but more drastic effects on protein properties. Theoretically, it is thought that a high mutational incidence of class (i) mutations is a result of lower likelihood of evolutionary purging when compared to class (ii) mutations, which is based on the structural and functional effects imparted at the protein level. Mutations from each class were also seen to co-occur as haplotypes, where they are thought to compensate for each other in terms of protein fitness [80].

**Fig. 2:**
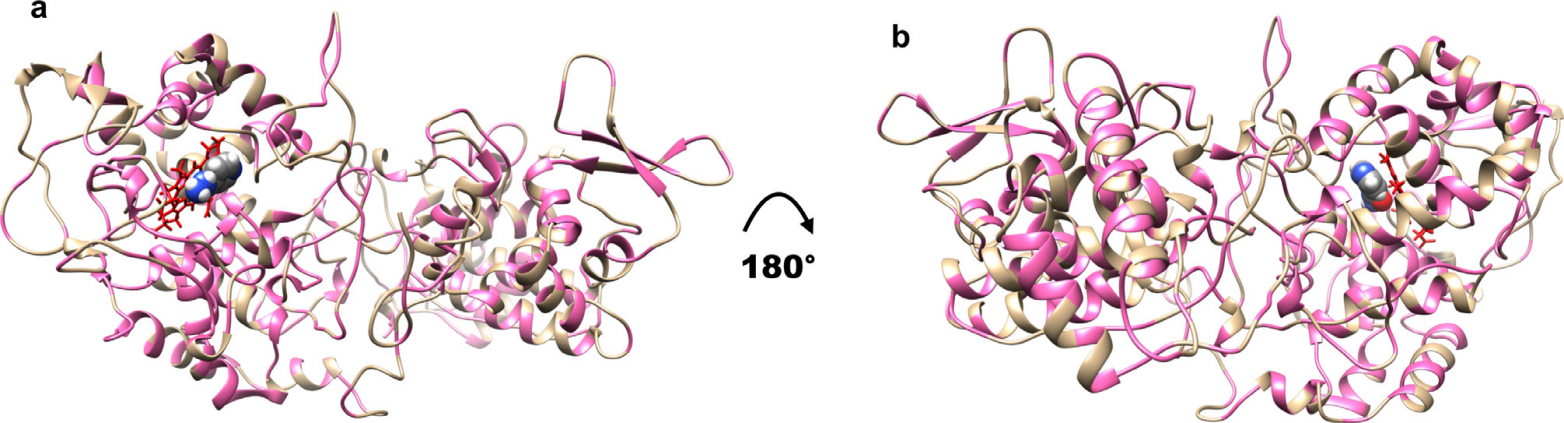
Structure of katG in complex with the drug isoniazid (INH) coloured by 378 mutational positions linked to 571 SNPs. Areas marked in pink are associated with one or more mutations. HEM is denoted in red, INH is denoted as spheres. Parts a) and b) denote the structure in two different orientations, rotated by 180°. Figure rendered using UCSF Chimera, Version 1.13.1. (For interpretation of the references to colour in this figure legend, the reader is referred to the web version of this article.)

**Fig. 3:**
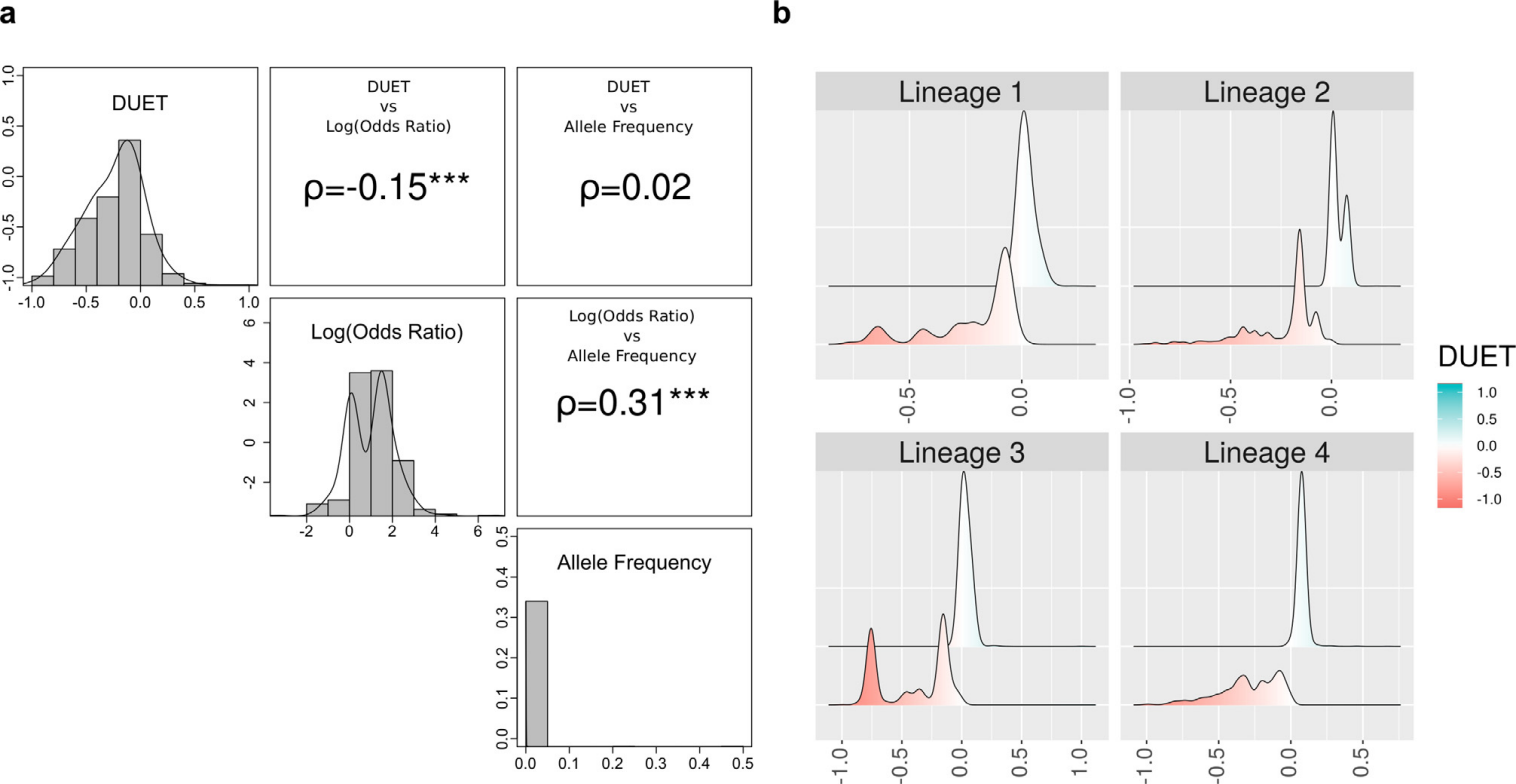
Relationship between the impact of *katG* mutations on Protein stability (DUET) with Odds Ratio (OR), Allele Frequency (AF) and *Mtb* lineages. **a)** Pairwise correlations between DUET protein stability and GWAS measures of OR and AF of 566 mutations (total number of mutations with associated OR). The upper panel in both plots include the pairwise Spearman correlation values (denoted by ρ) along with their statistical significance (***P < 0.001). **b)** Lineage distribution of samples with *katG* mutations showing *Mtb* lineages 1–4 according to DUET protein stability ranging from red (-1, most destabilising) to blue (+1, most stabilising). The number of samples within each lineage are: Lineage 1 (n = 2448), Lineage 2 (n = 6813), Lineage 3 (n = 5020) and Lineage 4 (n = 2739). The number of samples contribute to the 566 *katG* mutations. Figure generated using R statistical software, version 3.6.1. (For interpretation of the references to colour in this figure legend, the reader is referred to the web version of this article.)

Using 571 missense SNPs in *katG* across 19265 *Mtb* isolates, we tested for an association between mutation odds ratio and allele frequencies with the biophysical effect on protein stability **(**Fig. 2**).** This analysis suggests a higher proportion of destabilising mutations (~84%, n = 480 vs ~55.5%, n = 105) with only a small proportion of mutations lying within 10 Å of the active site (~10%, n = 57 vs ~15%, n = 28) highlighting the importance of allosteric mutations in INH drug resistance. There is a weak negative correlation between protein stability and odds ratio (ρ = −0.15, P < 0.001), and between protein stability and allele frequency (ρ = 0.31, P < 0.001) **(**Fig. 3**a)**. Analysis of biophysical effects (destabilising vs stabilising) of *katG* mutations by *Mtb* lineage revealed statistically significant differences **(**Fig. 3**b,** Kolmogorov-Smirnov *P* ≤ 1.3e-08**)**.

This type of analysis can be implemented on proteins encoded on plasmids (a common vector of resistance), where this approach has been used to explain the evolution of carbapenem resistance in *Acinetobacter baumannii* [91].

## 4 Computational structural tools predicting drug resistance

A limitation of current genomic sequencing-based resistance diagnostic approaches is that they require pre-existing knowledge about the phenotypic consequences of a variant. This means we often cannot detect it until it has been established within the population. By contrast, we have shown that using these tools we can pre-emptively identify likely drug resistant mutations in the absence of previous genomic data. These insights are of particular relevance for new drugs without extensive clinical data, and drugs which lack approved diagnostic tests. We have therefore used this approach to explore resistance against the TB drugs BDQ [81] and PZA [82]. The use of our PZA predictive model within the clinic was the first successful translational application of structural guided resistance detection. This revealed the power of combining structural interpretation within existing diagnostic sequencing frameworks [93]. Additionally, other ML based approaches have also been used in predicting drug resistance in *Mtb* [56], [138].

## 5 Designing better antibacterial drugs

It has been suggested that a way to minimise the development of resistance is by making compounds that interact similarly to a natural ligand [139]. The rationale being that this would lead to any resistance hot-spot having a higher fitness cost associated with it. This led to one of the first successful structure-guided drug discovery projects on neuraminidase inhibitors. Computational tools aid molecular characterisation of novel genomic variants, which provide opportunities to pre-empt likely resistant mutations. Anticipating these variants before they arise in a population can inform the drug discovery pipeline, especially in developing compounds less prone to resistance emergence. Such an approach has already been used as part of the drug development efforts against the TB drug target IMPDH [99]. The mutation predicted was the only resistant variant detected in subsequent in vitro resistant assays. Further, compounds designed to avoid this hot-spot were less prone to develop resistance [96], [97], [98]. This type of analysis complements the development of new tools that integrate genomic and structural data such as the Target-Pathogen online resource [140], which prioritises candidate drug targets in ten clinically important and diverse pathogens. This approach underscores the importance of structural data in guiding the drug-discovery process [140].

## 6 Summary and outlook

Large scale genomic studies have enabled identification of mutational associations with a resistance phenotype, useful for surveying the presence and spread of resistance to a wide range of antimicrobials. However, understanding the functional effects of putative mutations is crucial. Computational tools accounting for anti-symmetric properties of variation i.e. ΔΔG (A->B) = - ΔΔG (B->A) [118], [141], [142] are able to achieve improved prediction performance complementing experimental studies [85].

Genomic and structural analysis of resistance can infer mutational effects with therapeutic consequences before they become fixed in a pathogen population. This has implications for both infection surveillance and in the development of next generation drugs. The latter is of particular relevance to fragment-based drug discovery (FBDD) [143], [144]. For the past 20 years, this has been a powerful route to new therapeutics, for example, in the development of vemurafenib for late-stage melanoma [145], and is increasingly being applied in the search for new antimicrobial drugs [146], [147], [148]. FBDD uses a library of low molecular weight, low affinity binding molecules (fragments) to probe a target protein. This approach helps to identify areas that are receptive to binding. Biophysical and structural biology techniques are used to determine which fragment binds, and how. The target can then be used to guide an expansion of the fragment to a higher molecular weight and higher affinity binding molecule. An important step in this process is elaborating fragments that bind, to generate compounds that can be taken through to clinical testing. This is the stage at which crucial decisions are made about the regions of the drug target to exploit. However, pathogen tolerance is seldom considered, with direct consequences on drug effectiveness or efficacy. Current methods of analysing the effects of mutations either operate at the gene level (identifying known markers of resistance) or focus on a specific effect of the mutation (protein stability) without directly relating it to a resistance phenotype. Combining genomic results with structural analysis permits consideration of mutational impact on a potential drug binding region, providing informed decisions regarding drug efficacy. This has the potential to help the design of better antimicrobial drugs.

## CRediT authorship contribution statement

**Tanushree Tunstall:** Conceptualization, Formal analysis, Visualization, Writing - original draft. **Stephanie Portelli:** Conceptualization, Visualization, Writing - original draft. **Jody Phelan:** Data curation. **Taane G. Clark:** Writing - review & editing. **David B. Ascher:** Conceptualization, Supervision, Writing - review & editing. **Nicholas Furnham:** Conceptualization, Supervision, Writing - review & editing.

## Declaration of Competing Interest

The authors declare that they have no known competing financial interests or personal relationships that could have appeared to influence the work reported in this paper.
